# Supplementary material for: A Critical View of the Application of the APEX Software (Aqueous Photochemistry of Environmentally-Occurring Xenobiotics) to Predict Photoreaction Kinetics in Surface Freshwaters
Source: Molecules. 2019 Dec 18;25(1):9. doi: 10.3390/molecules25010009 (PMC7017383; doi:10.3390/molecules25010009)
Supplement: Supplementary file 1 [file molecules-25-00009-s001.zip › 3.2.4_gcc-4.4.0.zip!/3.2.4_gcc-4.4.0/3.2.4_gcc-4.4.0/doc/octave/html/interpreter/Loading-and-Saving-Images.html]

Loading and Saving Images - Untitled


Next: Displaying Images,
Up: Image Processing


---

### 31.1 Loading and Saving Images

The first step in most image processing tasks is to load an image
into Octave. This is done using the `imread` function, which uses the
`GraphicsMagick` library for reading. This means a vast number of image
formats is supported. The `imwrite` function is the corresponding function
for writing images to the disk.

In summary, most image processing code will follow the structure of this code

```
     I = imread ("my_input_image.img");
     J = process_my_image (I);
     imwrite ("my_output_image.img", J);
```

— Function File: [img, map, alpha] = **imread** (filename)  
> Read images from various file formats.
>
> The size and numeric class of the output depends on the
> format of the image. A color image is returned as an
> MxNx3 matrix. Grey-level and black-and-white images are
> of size MxN.
> The color depth of the image determines the numeric
> class of the output: "uint8" or "uint16" for grey
> and color, and "logical" for black and white.
>
>
>
> **See also:** imwrite, imfinfo.

— Function File: **imwrite** (img, filename, fmt, p1, v1, ...)  
— Function File: **imwrite** (img, map, filename, fmt, p1, v1, ...)  
> Write images in various file formats.
>
> If fmt is missing, the file extension (if any) of
> filename is used to determine the format.
>
> The parameter-value pairs (p1, v1, ...) are optional. Currently
> the following options are supported for JPEG images
>
> Quality: Sets the quality of the compression. The corresponding value should be an integer between 0 and 100, with larger values meaning higher visual quality and less compression.
>
>
>
>
> **See also:** imread, imfinfo.

— Built-in Function: val = **IMAGE\_PATH** ()  
— Built-in Function: old\_val = **IMAGE\_PATH** (new\_val)  
> Query or set the internal variable that specifies a colon separated
> list of directories in which to search for image files.

It is possible to get information about an image file on disk, without actually
reading it into Octave. This is done using the `imfinfo` function which
provides read access to many of the parameters stored in the header of the image
file.

— Function File: info = **imfinfo** (filename)  
— Function File: info = **imfinfo** (url)  
> Read image information from a file.
>
> `imfinfo` returns a structure containing information about the image
> stored in the file filename. The output structure contains the
> following fields.
>
> Filename: The full name of the image file. FileSize: Number of bytes of the image on disk FileModDate: Date of last modification to the file. Height: Image height in pixels. Width: Image Width in pixels. BitDepth: Number of bits per channel per pixel. Format: Image format (e.g., `"jpeg"`). LongFormat: Long form image format description. XResolution: X resolution of the image. YResolution: Y resolution of the image. TotalColors: Number of unique colors in the image. TileName: Tile name. AnimationDelay: Time in 1/100ths of a second (0 to 65535) which must expire before displaying the next image in an animated sequence. AnimationIterations: Number of iterations to loop an animation (e.g., Netscape loop extension) for. ByteOrder: Endian option for formats that support it. Is either `"little-endian"`, `"big-endian"`, or `"undefined"`. Gamma: Gamma level of the image. The same color image displayed on two different workstations may look different due to differences in the display monitor. Matte: `true` if the image has transparency. ModulusDepth: Image modulus depth (minimum number of bits required to support red/green/blue components without loss of accuracy). Quality: JPEG/MIFF/PNG compression level. QuantizeColors: Preferred number of colors in the image. ResolutionUnits: Units of image resolution. Is either `"pixels per inch"`, `"pixels per centimeter"`, or `"undefined"`. ColorType: Image type. Is either `"grayscale"`, `"indexed"`, `"truecolor"`, or `"undefined"`. View: FlashPix viewing parameters.
>
>
>
>
> **See also:** imread, imwrite.
